# Supplementary figures and images for: Investigation of the active ingredients and pharmacological mechanisms of Porana sinensis Hemsl. Against rheumatoid arthritis using network pharmacology and experimental validation
Source: PLoS One. 2022 Mar 2;17(3):e0264786. doi: 10.1371/journal.pone.0264786 (PMC8890728; doi:10.1371/journal.pone.0264786)

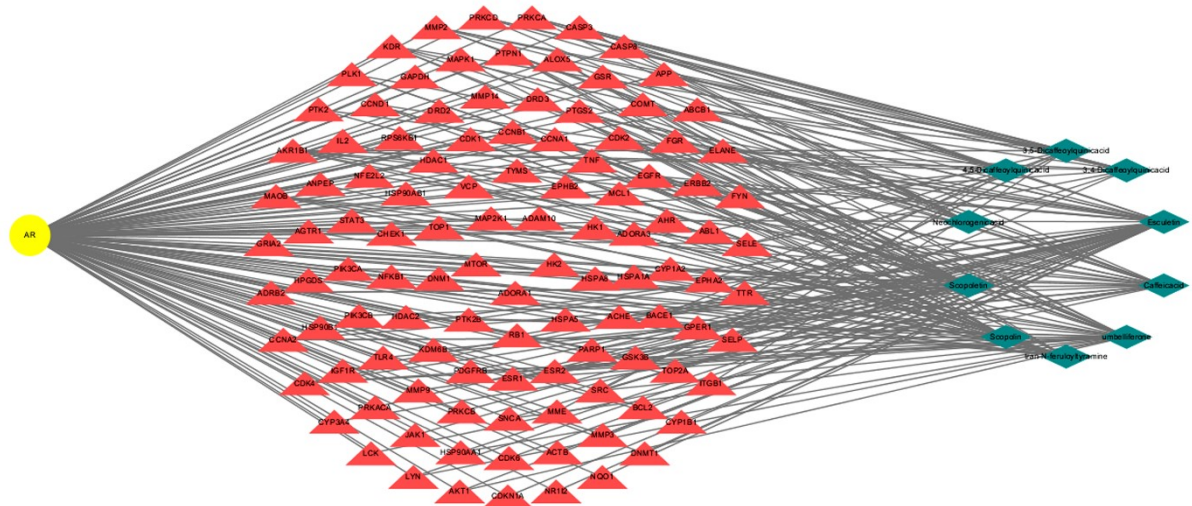

**S1 Fig. The C-T-D network for *P. sinensis* in treatment of rheumatoid arthritis**

Supplement: S1 Fig — (PDF) [file pone.0264786.s001.pdf]

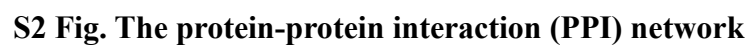

**S2 Fig. The protein-protein interaction (PPI) network**

Supplement: S2 Fig — (PDF) [file pone.0264786.s002.pdf]

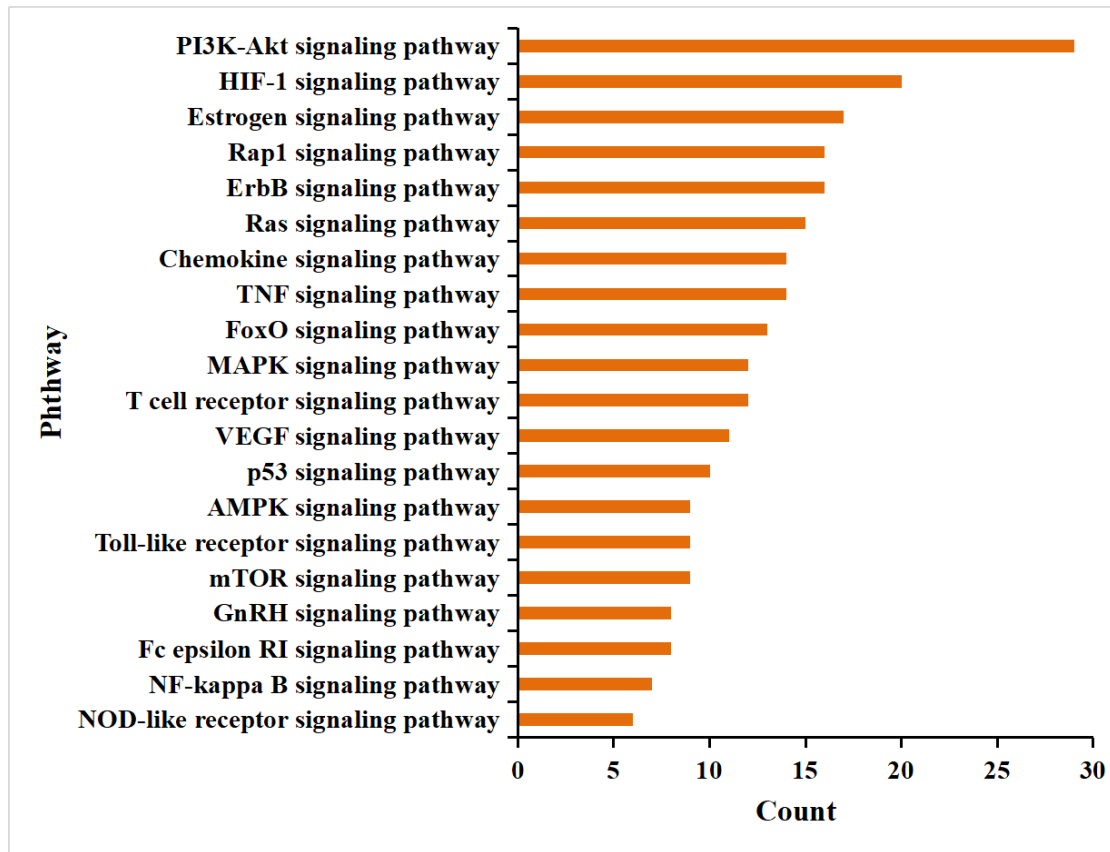

S3 Fig. The KEGG pathway for *P. sinensis* against rheumatoid arthritis

Supplement: S3 Fig — (PDF) [file pone.0264786.s003.pdf]

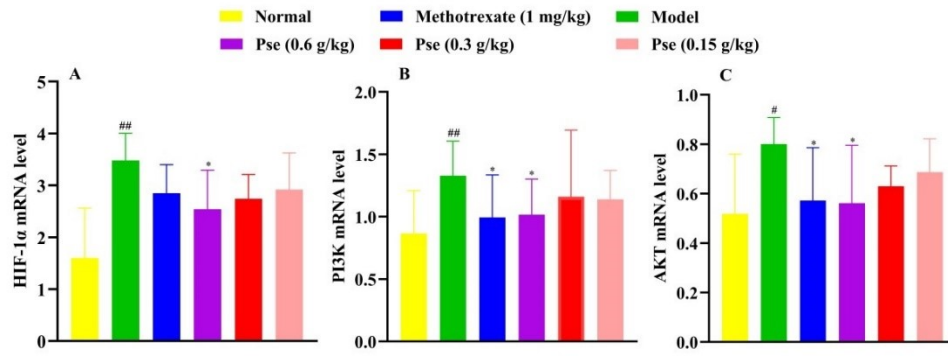

**S7 Fig. Effects of *P. sinensis* extract (Pse) on mRNA levels of HIF-1 $\alpha$  (A), PI3K (B) and AKT (C).**

Supplement: S7 Fig — Effects of P. sinensis extract (Pse) on mRNA levels of HIF-1α (A), PI3K (B) and AKT (C). (PDF) [file pone.0264786.s007.pdf]
