# Supplementary material for: Investigation of the active ingredients and pharmacological mechanisms of Porana sinensis Hemsl. Against rheumatoid arthritis using network pharmacology and experimental validation
Source: PLoS One. 2022 Mar 2;17(3):e0264786. doi: 10.1371/journal.pone.0264786 (PMC8890728; doi:10.1371/journal.pone.0264786)
Supplement: S4 Fig — (PDF) [file pone.0264786.s004.pdf]

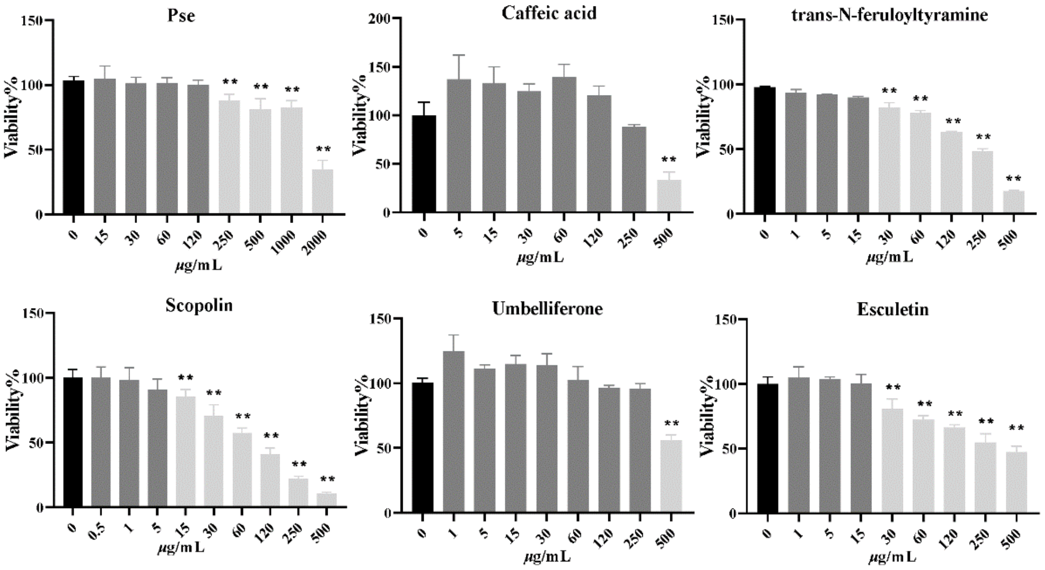

S4 Fig. Effects of cytotoxicity on RAW264.7 cells of *P. sinensis* extract (Pse) and its effective constituents ($^{**}P < 0.01$).
