# Supplementary material for: Investigation of the active ingredients and pharmacological mechanisms of Porana sinensis Hemsl. Against rheumatoid arthritis using network pharmacology and experimental validation
Source: PLoS One. 2022 Mar 2;17(3):e0264786. doi: 10.1371/journal.pone.0264786 (PMC8890728; doi:10.1371/journal.pone.0264786)
Supplement: S5 Fig — Effects of P. sinensis extract (Pse) on ankle circumference (A), arthritis index (B) and body weight (C) of rats. Values shown are mean ± SD (n = 8); *P < 0.05 and **P < 0.01 compared with model group; #P < 0.05 and ##P < 0.01 compared with normal group. (PDF) [file pone.0264786.s005.pdf]

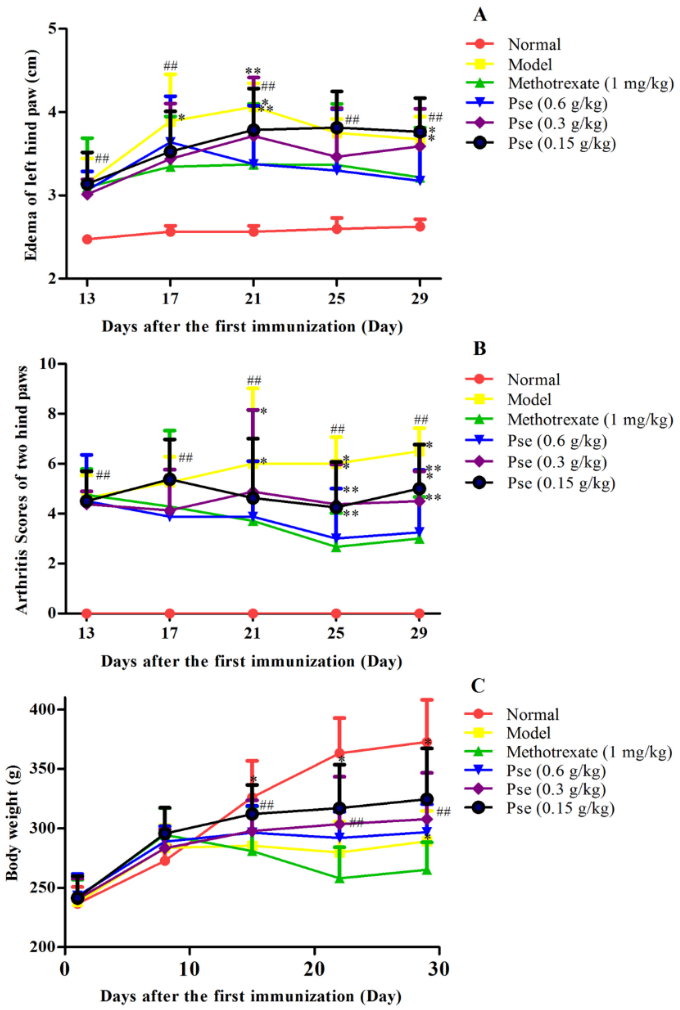

S5 Fig. Effects of *P. sinensis* extract (Pse) on ankle circumference (A), arthritis index (B) and body weight (C) of rats. Values shown are mean ± SD (n = 8); $^{*}P$ < 0.05 and $^{**}P$ < 0.01 compared with model group; $^{\#}P$ < 0.05 and $^{\#\#}P$ < 0.01 compared with normal group.
