# Supplementary material for: Investigation of the active ingredients and pharmacological mechanisms of Porana sinensis Hemsl. Against rheumatoid arthritis using network pharmacology and experimental validation
Source: PLoS One. 2022 Mar 2;17(3):e0264786. doi: 10.1371/journal.pone.0264786 (PMC8890728; doi:10.1371/journal.pone.0264786)
Supplement: S1 Table — (DOC) [file pone.0264786.s008.doc]

**S1 Table. Summary of gene-specific real-time PCR primer sequences**

| **Description** | **Gene bank** | **Sense primer** **(5ʹ→3ʹ)** | **Anti-sense primer (5ʹ→3ʹ)** |
| --- | --- | --- | --- |
| HIF-1α  PI3K | NM_024359.1  NM_001371300.1 | ACCGTGCCCCTACTATGTCG  TGACAGGCACAACGACAACATC | GCCTTGTATGGGAGCATTAACTT  AGGTAAGCCCTAACGCAGACAT |
| AKT | NM_033230.2 | CTGGAGGACAACGACTATGGC | AGCCTCTGTGTAGGGTCCTTCTT |
| r GAPDH | NM_017008.4 | CTGGAGAAACCTGCCAAGTATG | GGTGGAAGAATGGGAGTTGCT |
