# Supplementary material for: Investigation of the active ingredients and pharmacological mechanisms of Porana sinensis Hemsl. Against rheumatoid arthritis using network pharmacology and experimental validation
Source: PLoS One. 2022 Mar 2;17(3):e0264786. doi: 10.1371/journal.pone.0264786 (PMC8890728; doi:10.1371/journal.pone.0264786)
Supplement: S2 Table — (DOC) [file pone.0264786.s009.doc]

**S2 Table. The information of top 20 significant KEGG enrichment analysis (David, *P*-value＜0.05**)

| **ID** | **Description** | **GeneRatio** | ***P*-value** | **Count** | **Genes** |
| --- | --- | --- | --- | --- | --- |
| ptr04066 | HIF-1 | 0.11 | 1.97E-17 | 20 | MAP2K1, CDKN1A, PRKCB, STAT3, PRKCA, PIK3CB, HK2, EGFR, NFKB1, MTOR, IGF1R, HK1, RPS6KB1, PIK3CA, ERBB2, BCL2, AKT1, MAPK1, TLR4, GAPDH |
| ptr04151 | PI3K-Akt | 0.16 | 1.98E-14 | 29 | ITGB1, GSK3B, CDKN1A, HSP90AB1, PIK3CB, EGFR, HSP90B1, IGF1R, CCND1, KDR, AKT1, MAPK1, JAK1, MCL1, PDGFRB, HSP90AA1, MAP2K1, NFKB1, MTOR, PTK2, IL2, CDK6, RPS6KB1, PIK3CA, CDK4, CDK2, BCL2, TLR4, EPHA2 |
| ptr04915 | Estrogen | 0.09 | 2.55E-13 | 17 | HSPA8, HSP90AA1, MAP2K1, HSP90AB1, SRC, MMP2, PRKCD, PIK3CB, ESR1, MMP9, ESR2, EGFR, HSP90B1, PIK3CA, AKT1, MAPK1, PRKACA |
| ptr04012 | ErbB | 0.09 | 8.85E-13 | 16 | GSK3B, MAP2K1, CDKN1A, PRKCB, SRC, PRKCA, PIK3CB, PTK2, EGFR, MTOR, RPS6KB1, PIK3CA, ERBB2, ABL1, AKT1, MAPK1 |
| ptr04668 | TNF | 0.08 | 3.47E-09 | 14 | MAP2K1, MMP3, PIK3CB, PTGS2, TNF, MMP9, SELE, NFKB1, MMP14, CASP8, PIK3CA, CASP3, AKT1, MAPK1 |
| ptr04370 | VEGF | 0.06 | 2.48E-08 | 11 | MAP2K1, PIK3CA, SRC, PRKCB, KDR, AKT1, MAPK1, PRKCA, PIK3CB, PTGS2, PTK2 |
| ptr04660 | T cell receptor | 0.06 | 2.58E-07 | 12 | GSK3B, MAP2K1, PIK3CA, LCK, CDK4, AKT1, MAPK1, FYN, PIK3CB, TNF, IL2, NFKB1 |
| ptr04068 | FoxO | 0.07 | 3.21E-07 | 13 | MAP2K1, CDKN1A, PLK1, STAT3, PIK3CB, EGFR, IGF1R, CCNB1, CCND1, PIK3CA, CDK2, AKT1, MAPK1 |
| ptr04015 | Rap1 | 0.09 | 3.74E-07 | 16 | PDGFRB, ITGB1, MAP2K1, PRKCB, SRC, PRKCA, PIK3CB, EGFR, ACTB, IGF1R, PIK3CA, KDR, AKT1, MAPK1, DRD2, EPHA2 |
| ptr04115 | p53 | 0.06 | 7.16E-07 | 10 | CDKN1A, CCNB1, CDK6, CASP8, CCND1, CDK4, CASP3, CHEK1, CDK2, CDK1 |
| ptr04062 | Chemokine | 0.08 | 1.33E-06 | 14 | LYN, GSK3B, MAP2K1, SRC, STAT3, PIK3CB, PTK2, NFKB1, FGR, PIK3CA, AKT1, PTK2B, MAPK1, PRKACA |
| ptr04150 | mTOR | 0.05 | 1.59E-06 | 9 | PIK3CA, RPS6KB1, PRKCB, AKT1, MAPK1, PRKCA, PIK3CB, TNF, MTOR |
| ptr04014 | Ras | 0.08 | 5.34E-06 | 15 | PDGFRB, MAP2K1, PRKCB, PRKCA, PIK3CB, EGFR, NFKB1, IGF1R, PIK3CA, KDR, ABL1, AKT1, MAPK1, PRKACA, EPHA2 |
| ptr04664 | Fc epsilon RI | 0.04 | 6.92E-05 | 8 | LYN, MAP2K1, PIK3CA, AKT1, MAPK1, FYN, PIK3CB, TNF |
| ptr04620 | Toll-like receptor | 0.05 | 1.15E-04 | 9 | MAP2K1, CASP8, PIK3CA, AKT1, MAPK1, PIK3CB, TLR4, TNF, NFKB1 |
| ptr04152 | AMPK | 0.05 | 3.32E-04 | 9 | CCNA2, CCNA1, CCND1, PIK3CA, RPS6KB1, AKT1, PIK3CB, MTOR, IGF1R |
| ptr04912 | GnRH | 0.04 | 4.33E-04 | 8 | MAP2K1, MMP14, SRC, MMP2, PTK2B, MAPK1, PRKACA, EGFR |
| ptr04621 | NOD-like receptor | 0.03 | 0.001058239 | 6 | HSP90AA1, HSP90AB1, CASP8, MAPK1, TNF, NFKB1 |
| ptr04012 | MAPK | 0.07 | 0.001193495 | 12 | PDGFRB, HSPA8, MAP2K1, PRKCB, CASP3, AKT1, MAPK1, PRKCA, PRKACA, TNF, EGFR, NFKB1 |
| ptr04621 | NF-kappa B | 0.04 | 0.001892095 | 7 | LYN, LCK, BCL2, PTGS2, TLR4, TNF, NFKB1 |
