# Supplementary material for: Investigation of the active ingredients and pharmacological mechanisms of Porana sinensis Hemsl. Against rheumatoid arthritis using network pharmacology and experimental validation
Source: PLoS One. 2022 Mar 2;17(3):e0264786. doi: 10.1371/journal.pone.0264786 (PMC8890728; doi:10.1371/journal.pone.0264786)
Supplement: S1 File — (ZIP) [file pone.0264786.s010.zip › Supporting Information - CompressedZIP File Archive/S1_raw_images.pdf]

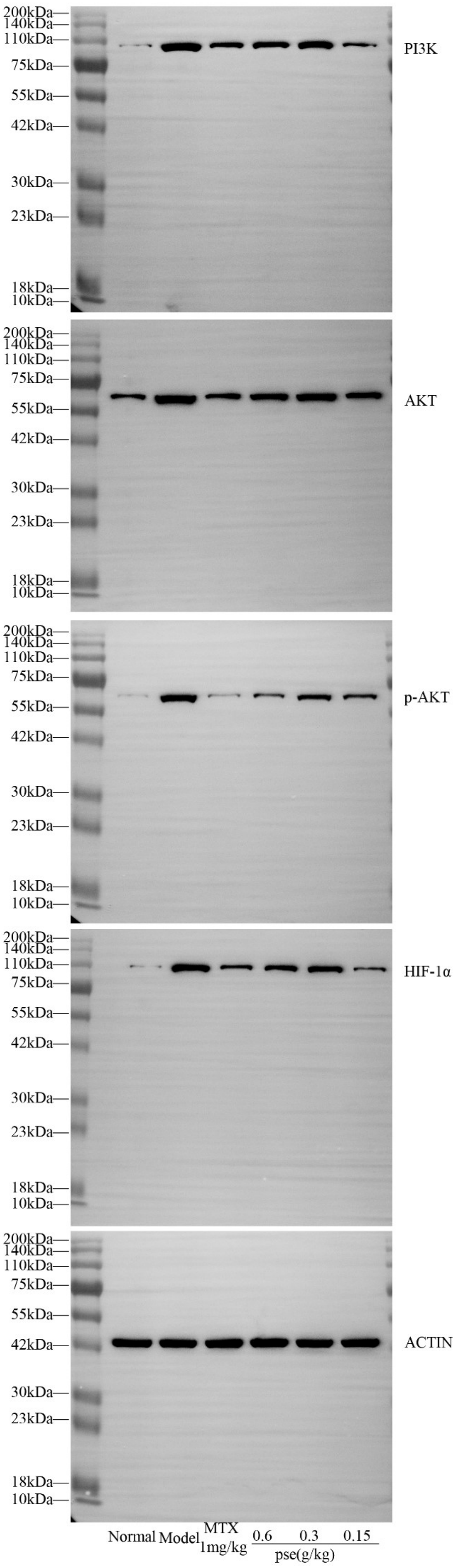
200kDa
140kDa
110kDa
75kDa
55kDa
42kDa
30kDa
23kDa
18kDa
10kDa
PI3K
AKT
p-AKT
HIF-1α
ACTIN
Normal
Model
MTX
1mg/kg
0.6
0.3
0.15
pse(g/kg)

Effects of *P. sinensis* extract (Pse) on the expression levels of PI3K, AKT, p-AKT, and HIF-1α proteins. Protein visualization was conducted on an imaging system.
