# Supplementary material for: Investigation of the active ingredients and pharmacological mechanisms of Porana sinensis Hemsl. Against rheumatoid arthritis using network pharmacology and experimental validation
Source: PLoS One. 2022 Mar 2;17(3):e0264786. doi: 10.1371/journal.pone.0264786 (PMC8890728; doi:10.1371/journal.pone.0264786)
Supplement: Attachment — Submitted filename: Response to Reviewers.doc [file pone.0264786.s012.doc]

**Manuscript Reference Number: PONE-D-21-26996R1**
**Manuscript Title:** Investigation of the active ingredients and pharmacological mechanisms of *Porana sinensis* Hemsl. against rheumatoid arthritis using network pharmacology and experimental validation
**Journal:** PLOS ONE

**1. According to the new policies of PLoS One, authors should provide uncropped gels and blots. We have asked to provide this information, but now the raw data do not show the whole gels and blots. Please provide them.**

**Response:** Thanks for your suggestion. We have provided uncropped gels and blots in S1_raw_images (pdf) in supporting information.

**2. The cytotoxicity asked has not been fulfilled. Instead, the cytotoxicity of individual compounds has been included and not the extracts as mentioned.**

**Response:** Thanks for your suggestion. The cytotoxicity of *P. sinensis* extract (Pse) has been provided in S4 Fig in supporting information.
