# Supplementary material for: Investigation of the active ingredients and pharmacological mechanisms of Porana sinensis Hemsl. Against rheumatoid arthritis using network pharmacology and experimental validation
Source: PLoS One. 2022 Mar 2;17(3):e0264786. doi: 10.1371/journal.pone.0264786 (PMC8890728; doi:10.1371/journal.pone.0264786)
Supplement: Attachment — Submitted filename: Response to Reviewers.doc [file pone.0264786.s013.doc]

**Manuscript Reference Number: PONE-D-21-26996R2**
**Manuscript Title:** Investigation of the active ingredients and pharmacological mechanisms of *Porana sinensis* Hemsl. against rheumatoid arthritis using network pharmacology and experimental validation
**Journal:** PLOS ONE

**1. We have sent two request to be addressed. The cropped gels are still an issue. You have provided the immunoblots already cropped from the original gel. The request is to show the original gel that was used to crop.**

**Response:** Thanks for your suggestion. We have provided the original gel in S1_raw_images (pdf) in supporting information.
